# Patients’ experiences of changes in health complaints before, during, and after removal of dental amalgam

**DOI:** 10.3402/qhw.v10.28157

**Published:** 2015-06-24

**Authors:** Therese T. Sjursen, Per-Einar Binder, Gunvor B. Lygre, Vigdis Helland, Knut Dalen, Lars Björkman

**Affiliations:** 1 Department of Clinical Dentistry, Faculty of Medicine and Dentistry, University of Bergen, Bergen, Norway; 2 Department of Clinical Psychology, Faculty of Psychology, University of Bergen, Bergen, Norway; 3 Dental Biomaterials Adverse Reaction Unit, Uni Research, Bergen, Norway; 4 Department of Biological and Medical Psychology, Faculty of Psychology, University of Bergen, Bergen, Norway

**Keywords:** Health, health seeking, illness experiences, dental amalgam, assigning meaning, reflexivity, thematic analysis

## Abstract

In this article, we explore how patients with health complaints attributed to dental amalgam experienced and gave meaning to changes in health complaints before, during, and after removal of all amalgam fillings. We conducted semistructured qualitative interviews with 12 participants from the treatment group in a Norwegian amalgam removal trial. Interviews took place within a couple months of the final follow-up 5 years after amalgam removal. Using the NVivo9 software, we conducted an explorative and reflective thematic analysis and identified the following themes: Something is not working: betrayed by the body, You are out there on your own, Not being sure of the importance of amalgam removal, The relief experienced after amalgam removal, and To accept, to give up, or to continue the search. We discuss the findings in the context of patients’ assigning meaning to illness experiences.

The meanings given to symptoms and distress can transform suffering. Meaning—*any* meaning—serves to turn back the tide of chaos and bafflement that confronts us in affliction. Given specific meaning, illness becomes metaphor—a rhetorical resource to be used to explore and communicate the wider significance of our predicament. (Kirmayer, 1994, p. 183)

Patients suffering from health complaints which cannot be fully explained by the doctors’ findings might find it difficult to assign meaning to their illness experiences (Kornelsen, Atkins, Brownell, & Woollard, 2015; Madden & Sim, 2006). How can they understand the experienced pain and discomfort when the biomedical “stamp of approval”—a diagnosis—is apparently not within reach? How can they justify not being able to partake in activities as they did previously when their suffering remains unconfirmed by the medical system?

It is well known that mercury vapor released from amalgam fillings can be inhaled and absorbed into the bloodstream (Clarkson, Magos, & Myers, 2003). Some patients fear their health complaints might be caused or aggravated by mercury released from their amalgam fillings (Sjursen et al., 2014; Tillberg et al., 2005). Patients who attribute health complaints to their dental amalgam fillings are a heterogeneous group. Common to all of them is that they suffer from unexplained or partially explained health complaints that they believe are caused or aggravated by their amalgam fillings. For some, only one or a few local complaints such as taste disturbances, dry mouth, and intraoral pain are attributed to the dental amalgam. The majority describe a number of both local and general health complaints involving several organ systems. Tiredness, headaches, pain in muscles and joints, and problems with memory and concentration are among the most frequently reported complaints (Langworth, Björkman, Elinder, Järup, & Savlin, 2002; Lygre, Gjerdet, & Björkman, 2005; Vamnes, Lygre, Grönningsæter, & Gjerdet, 2004). For some patients, contact allergic reactions might be present, and removal of amalgam fillings in contact with such lesions is generally recommended (Issa, Brunton, Glenny, & Duxbury, 2004; Lygre, Gjerdet, & Björkman, 2004). For the majority of patients with health complaints attributed to dental amalgam, no objective signs of adverse reactions can be observed (Langworth et al., 2002; Vamnes et al., 2004). Nevertheless, one cannot preclude the possibility that dental amalgam could have deleterious effects on the health of highly susceptible people (Needleman, 2006; US Food and Drug Administration, 2009). This poses the following dilemma: Even though there is not sufficient evidence to lend scientific credibility to an amalgam syndrome diagnosis, standard safety margins are lacking, thus making it impossible to rule out that, for some people, amalgam might be associated with a risk of negative health effects (Richardson et al., 2011).

Studies investigating changes in general health complaints after removal of amalgam fillings have found reductions in health complaints (Lygre et al., 2005; Melchart et al., 2008; Nerdrum et al., 2004; Sjursen et al., 2011), but not to the levels found in the general population (Lygre et al., 2005; Nerdrum et al., 2004). The observed reductions in health complaints might be interpreted as effects of patients being seen and heard, improved dental conditions, the natural variation in the course of the complaints, reduced exposure to mercury, as well as placebo effect and discontinued nocebo effect (Melchart et al., 2008; Nerdrum et al., 2004; Sjursen et al., 2011). Because of difficulties in masking whether patients have their amalgam fillings removed or not, randomized clinical trials of the effects of amalgam removal are likely to be influenced by participants’ expectations.

In previous studies, patients’ experiences have seldom been explored on their own terms. In a focus group study from New Zealand (Jones, 2004) with 35 participants having amalgam-related complaints, participants described experiencing psychological problems such as memory loss and mood swings that they believed were related to their amalgam fillings. They also described experiencing psychological problems, such as loss of social support and considering suicide, that they related to suffering from symptoms that were not easily diagnosed and thereby often treated as indicating hypochondriac tendencies. Of the participants who had removed all amalgam fillings, the majority reported improved health; some even to the extent of full recovery (Jones, 2004). In a Swedish interview study (Stahlnacke & Soderfeldt, 2013) of persons who attribute health problems to dental filling materials, mostly dental amalgam, the participants described a variety of long-lasting health problems that they believed were caused by dental amalgam. Replacement of dental materials was the main treatment for these problems, and the majority of the participants reported having had good experiences with health professionals, although some negative encounters were also reported (Stahlnacke & Soderfeldt, 2013).

When patients suffer from health complaints that cannot be easily explained, both patients and health personnel find themselves in a situation where the normal expectations of the medical encounter cannot be met. To be better able to meet the patient where he or she is, it is important that health personnel take the time to learn more about how patients interpret and give meaning to their health complaints. Patients experience and give meaning to health complaints in their everyday life, and it is therefore important to know how the patients’ thoughts, obligations, past experiences, and perceptions of the future interact with the perceived pain and discomfort. Consequently, for patients with health complaints attributed to dental amalgam, it is not only necessary to bridge the gap between the medical and dental aspects, it is also necessary to bridge the gap between how the complaints are understood in the physician's/dentist's office and how they are understood and experienced in the context of the patient's everyday life.

In a previous article (Sjursen et al., 2014), we explored how patients came to attribute their unexplained health complaints to dental amalgam. In this article, our aim is to explore how the same patients experienced and gave meaning to changes in health complaints before, during, and after amalgam removal.

## Method

### Participants

Participants were recruited from the intervention group in a Norwegian amalgam removal trial (Sjursen et al., 2011). To be eligible for participation in the intervention group of the trial, participants had to fulfill the following criteria: initially referred to a specialty unit for examination of health complaints attributed to dental amalgam; no signs of contact allergic reactions to dental amalgam and thereby not recommended for removal of amalgam fillings; amalgam fillings still present; health complaints from at least three organ systems; mercury level data available from initial examination; no allergy to resin-based dental materials; no need for complicated dental therapy; and no severe medical disorders/food allergies/psychological difficulties.

The 20 participants in the intervention group had all their amalgam fillings replaced with other restorative materials by their regular dentists. Amalgam fillings were removed according to guidelines ensuring minimal exposure from mercury (Dental Biomaterials Adverse Reaction Unit, 2002). The cost of the amalgam removal was covered by project funds for the amalgam removal trial. Follow-ups took place approximately 3 months and 1, 3, and 5 years after the participants had completed the removal of all their amalgam fillings. At the 5-year follow-up, 12 (seven women and five men) of the participants were invited to participate in qualitative research interviews. All accepted, and interviews were scheduled accordingly. At the time of the interviews, age range of the participants was from 45 to 65 years (mean age 54.4 years). After the completion of the 12 interviews, we were able to identify both convergent and divergent experiences in our data material. As we did not have the impression that the last interviews brought to light new themes, we decided to stop recruiting participants at this point.

### Sampling method

We used a purposive sampling procedure to recruit participants from the intervention group in an amalgam removal trial to explore how they experienced and gave meaning to changes in health complaints before, during, and after amalgam removal. By choosing this sampling procedure, we were able to obtain a homogenous sample with regard to all participants having had their amalgam fillings removed. When it came to the demographic characteristics, participants were selected to ensure that a diverse age range and both sexes were represented.

### Researchers

The interview study was carried out as a cross-disciplinary collaboration between three psychologists, two dentists, and one operating nurse. Together we have varied clinical experience, as well as a diverse experience with both qualitative and quantitative research methods.

### Data collection

To lay the basis for an open exploration of participants’ experiences of changes in health complaints and how they assigned meaning to these, we chose to carry out semistructured, exploratory, in-depth interviews. The first author, in close cooperation with the fifth author, carried out all interviews. Neither had been present at the follow-ups, and the interviews were held at a different location than the follow-ups. After each interview, the first and fifth author adjusted the interview guide that had been initially developed by all the authors. The interviews were videotaped. Mean duration of the interviews was 60 min (range 32 min to 2 h 9 min).

### Analysis

By reading and comparing the individual accounts, we wanted to identify similarities and discrepancies in the ways in which the participants experienced and gave meaning to changes in health complaints before, during, and after amalgam removal. We conducted an explorative and reflexive thematic analysis (Binder, Holgersen, & Moltu, 2012; Braun & Clarke, 2006), which can be summarized as follows: (a) the first author transcribed all interview recordings verbatim, (b) to get a basic sense of patterns in the participants’ experiences, all authors read through the written material separately, (c) to establish meaningful themes, each author discussed the material with the first author, (d) the first author organized the text material, with the assistance of the NVivo9 software (QSR International Pty Ltd., 2010), into “nodes” in accordance with these themes, (e) in cooperation with the coauthors, the themes were additionally refined and condensed into the presented findings, and (f) examples and quotes were selected to illustrate how patients experienced and gave meaning to changes in health complaints. To strengthen the transparency of the analysis, we presented thick descriptions and used quotes that exemplify the themes (Denzin, 2001; Geertz, 1973; Ponterotto, 2006).

### Ethical concerns

Participants received written and verbal information about the interviews at the time of the 5-year follow-up, and all included participants signed a consent form. Before they entered the interview room, the participants were reminded that the interviews were going to be videotaped. The Regional Committee for Medical and Health Research Ethics in Western Norway, and the Norwegian Social Science Data Services approved the study. To safeguard the anonymity of participants, findings are presented without identifying details.

### Findings

In our analyses of how patients experienced and gave meaning to changes in health complaints before, during, and after amalgam removal, we found the following themes to be of importance:

- Something is not working: betrayed by the body.
- You are out there on your own.
- Not being sure of the importance of amalgam removal.
- The relief experienced after amalgam removal.
- To accept, to give up, or to continue the search.

#### Something is not working: betrayed by the body

The starting point for all participants was the experience of something not working inside their bodies. Some had struggled with health complaints from an early age, whereas others experienced onset of complaints as adults. The majority of the participants described the onset of complaints as gradual, but some pinpointed more distinct starting points for the health complaints they attributed to dental amalgam. Several of the participants already had—or went on to receive—other diagnoses explaining part of their complaints; nevertheless, they felt that something remained unexplained. Participants’ complaints differed in kind, number, and intensity. The following complaints were mentioned most often: pain in muscles and joints, headaches, memory problems, tiredness, gastrointestinal symptoms, and intraoral health complaints. For some, the discomfort and impairment were limited to a few distinct complaints; for others, it was the sum of the complaints—more than the separate complaints in themselves—that posed the main burden. Some participants were puzzled by the way the complaints made them feel “beside themselves” or “out of it.”

I was in so much pain, and I also felt, for a while, that I had such a poor memory (sighs). I cannot say if that was because of stress caused by having to fight the pain, but I did feel “out of it” in a way. I really did.

Some described their bodies as being overly sensitive to many different things to a degree that some even felt betrayed by their bodies. They found it necessary to avoid certain foodstuffs, such as wheat and/or sugar, and some also developed respiratory reactions and headaches from certain odors such as perfume and paint. One participant described some of her puzzling complaints and asked, “What causes it? Why did it happen? Was it because of my strange body? Who knows?” Another participant seemed saddened that her body was not working as well as others’ appeared to function. Because of her complaints, she was only able to keep a part-time job, and even then, she often felt exhausted and in pain after work. Several described how the health complaints had negative consequences for their social life. They recounted the various ways the complaints and, in particular, the depleted energy levels and nausea caused by the pain limited their ability to keep up with family life and professional obligations. They felt they could not perform as well, or at least not as effortlessly, as others seemed to be able to do. Despite having families that gave them support and understanding, several described a profound feeling of sadness related to not being able to be the spouse/parent they wanted to be. Several also felt that their relationship with friends and colleagues suffered because of their complaints. They seldom had the energy to meet people socially, and when in pain, they had to pull themselves together to avoid responding more harshly than they wanted to in tense situations. All participants worked hard to ensure that they did not lash out and hurt the people around them, and most of the time they thought they succeeded with this. This was very important to all of them, and the occasional slip-up was not taken lightly.

If it only affected oneself, it would be more than terrible, but it gets even worse if it hurts others. And sometimes it ends up in a way that one is not able to be the person one would like to be.

It became important not only for them but also for the significant people in their lives, to search for a way to understand and hopefully cure the complaints.

#### You are out there on your own

The majority of the participants in our sample said that they had been actively trying to find explanation for their complaints. Several were disappointed by how little the medical profession had to offer when it came to health complaints in the absence of corresponding objective findings.

I'm not quite able to sort it out, and the doctors are not very good at helping with these things when they do not find anything specific…. So in a way, you have to sort it out on your own.

In addition to seeking help from physicians and dentists, participants also had consulted physiotherapists, chiropractors, and practitioners of alternative medicine. For some participants, this had yielded immediate and striking results, such as the case of one participant, who consulted a healer because of a locked temporomandibular joint.

Then I saw a healer for the first time, and I have never experienced anything so strange. I mean, he didn't even touch me, but it creaked and groaned and after that, I have been able to open my mouth wide.

A few of the participants who had consulted practitioners of alternative medicine had developed quite close relationships with some of them. In addition to the treatment *per se*, it seemed that these therapists filled an important role as emphatic listeners and givers of advice relating to many aspects of the participants’ lives. Other participants only sought treatment when they needed help to manage specific complaints. They tried to limit the number of treatment sessions as these were described as expensive and time consuming. There were also participants who had spent a considerable amount of time, energy, and money on treatments that were described as having from minor effect to no effect at all.

Participants had also made other changes in their lives, hoping to diminish their health complaints. Several had tried different diets, sometimes through trial-and-error, and other times on advice given at rehabilitation centers or by practitioners of alternative medicine. For most, the results were promising at first, but the beneficial changes did not last over time. Several participants, however, did continue to avoid or limit the intake of certain food types as they experienced this to be somewhat helpful. Most of the participants had also modified their work situation. Some had started working reduced hours, some had changed to jobs that were less physically taxing, and some had started saying “no” more often at work. One participant said that the questions the project's physician had asked her at the pretreatment examination led her to take a closer look at the way she was living her life, and she had realized that she needed to make more room for herself in her own life.

Participants varied as to how and when dental amalgam was suspected to be a possible cause for their body not working properly (Sjursen et al., 2014). When they first contacted the specialty unit, there was considerable media coverage of possible harmful effects of dental amalgam, and all participants acknowledged having heard about this possible connection through the media or through accounts from friends and acquaintances. In addition, they had all experienced something that made the link between dental amalgam and health complaints seem personally relevant. For some, dental amalgam ended up as the only plausible explanation remaining after they had tried everything else; for others, dental amalgam was thought to be only one of many factors influencing their health. Common to all participants was a strong desire to have the amalgam removed once the attribution of health complaints to dental amalgam was made.

#### Not being sure of the importance of amalgam removal

Participants said that they were very happy to be given the opportunity to have all amalgam fillings removed through participation in the clinical trial. Several pointed out that they would otherwise not have been able to afford such extensive dental treatment. Many of the participants emphasized that they had felt well taken care of both by their dentist and by the personnel at the specialty unit during follow-ups. To limit patients’ exposure to mercury, a protective sheet (rubber dam) made from silicone was used during amalgam removal. Several of the participants said this made them feel well-protected. A few patients had experienced illness episodes after treatment sessions. Two of the patients who had experienced adverse reactions said that they felt worse after treatment sessions when the rubber dam had been difficult or impossible to place.

When responding to the opening question: “Have you experienced any changes in health complaints or quality of life after the amalgam removal?” nine participants said that they had experienced changes for the better. One participant said she was unable to answer this question because she had been in a very demanding life situation at the time of the amalgam removal. Two men answered no to this question. They had both received other diagnoses and no longer suspected that dental amalgam was the cause of their complaints. The participants who had experienced changes for the better were somewhat hesitant when it came to identifying the amalgam removal as a direct cause for the changes. After they described the perceived changes in health complaints, they usually tried to sort out which changes they thought were caused by the amalgam removal and which were more likely to have been brought on by other changes in their lives.

Well, what I think is that I don't really know what (pause). I think that the amalgam removal at least has had an effect on my mouth and the pain I had there. But I (pause) when it comes to the other complaints, I think that it is kind of impossible to know if it is [the amalgam removal] that has made me better or if it is other things. I have tried a lot of different things. I have had different treatments, and I have changed my diet, you know, and I have started to take Omega-3 supplements, which is also supposed to be good for the joints, for instance. So, I really have done other things as well, and I really can't say if it is the teeth or if it is the other things or if it is (pause). I find this to be very difficult.

Participants thought that the new white fillings were much nicer looking than the old black fillings, and some of the participants said that they felt their oral condition had greatly improved after the amalgam removal. Two participants reported that a taste disturbance (metallic taste) had disappeared and they were reasonably certain that this was because the amalgam had been removed. One participant had to replace several of the new fillings due to new caries lesions. Participants found it easier to connect reduced intraoral health complaints, such as reduced pain and smarting in the gingiva, to the amalgam removal, than to connect the more general health complaints to the removal.

When it came to the general health complaints, all participants were quick to point out that both the initial complaints and the subsequent changes might have been influenced by changes in life situation, work conditions, and so forth. Several of the participants used phrases like “but, of course, this could also have been influenced by the stress caused by….” They also emphasized that they had been trying several treatment options both before and after the amalgam removal, and several of the women pointed to menopause as a possible explanation for reductions of some health complaints. Some of the participants had previously taken care of elderly parents, whereas other participants had this responsibility at the time of the interview. Some had gone through a divorce or a painful breakup after the amalgam removal and said that this had also influenced their health and general well-being. At the time of the interview, several participants were in demanding life situations that negatively affected their health, and several described how fluctuations of other medical conditions, both previously known and recently diagnosed, made it difficult to assess which changes were directly related to the amalgam removal.

#### The relief experienced after amalgam removal

Despite the uncertainties described in the last theme, the majority of the participants concluded that they were in a much better place in their lives at the time of the interview than they had been before the amalgam removal. With the exception of the two men who said they had experienced no changes in health complaints after amalgam removal, all participants believed that the amalgam removal was partially responsible for their feeling better.

This amalgam removal, I do believe it has had an effect, together with all the other things. But I would have to have psychic abilities to know exactly how. As I have told you, there are still periods in which I feel quite poorly and beside myself, but I do feel much better now. I really do.

All participants, including the participant who had experienced several new caries lesions after the removal, seemed relieved that they no longer had any amalgam fillings in their teeth. For many of the participants, this relief appeared to be associated with being able to cross a worry off a list.

Participant (P): Well, I was very relieved that I could have them removed…. Because, at that time, I was very focused on what was causing me to be not as healthy as others, and this was something I wanted to try to (pause) that it might help me get better. So it was certainly a plus to get rid of it. At least I did not have those anymore, and I had kind of excluded something (laughs). It was a little bit like that.

Interviewer (I): Yes, it felt good to

P: You know, some (pause). There are many people with the same complaints that I have had who are talking about amalgam and such. So it is possible that if I still had those fillings left, I could have been constantly thinking “Yes, it really could be those fillings keeping me from feeling well.” But it is not like that anymore, is it?

For almost all participants, there was a distinct change in emotionality and tone when asked how they would have felt if they still had one amalgam filling left. All responded that they would have had it removed and emphasized that they would not have been happy at all. This stood in stark contrast to the calm replies of some who had stated that they had never been totally sure of the connection between amalgam and health complaints to begin with, and who conveyed in other parts of the interview a quite sophisticated understanding of health as being multifactorially determined. This uncertainty related to the importance of the amalgam removal stood almost paradoxically in contrast to the absolute certainty, even 5 years after removal, that it was important to get rid of all amalgam fillings.

#### To accept, to give up, or to continue the search

Despite feeling better, as reported by the majority of the participants, none of them had become symptom-free after the amalgam removal. They reacted to this in different ways. For some, there seemed to be a change in the urgency to seek answers. A few even thought that they were moving toward accepting their health complaints, or at least toward accepting that their complaints could never be fully explained.

Well, in a way I have accepted that I will always have some complaints. I am not like I used to be when I thought that if only I could find the right solution, then I would also get cured. I have kind of given up on that. It is more about finding the best possible way to live with [the complaints].

For some participants, this was associated with growing older and accepting complaints as something to be expected with advancing age. For others, the acceptance seemed to be more a consequence of the limited success of previous attempts at finding answers. The quest for an answer comes at a cost, as reflected in the theme, “You are out there on your own.” In addition to the time and energy spent, there is also an emotional toll entailed in getting your hopes up and then being disappointed repeatedly. The process toward acceptance was described as containing both elements of relief, in that they could ease up on the search for an answer, and sadness at having to let go of their hope for a cure. One participant who suffered from daily pain and personal limitations caused by a diagnosed disease very firmly stated that she preferred a growth perspective to a pain-coping perspective. She did not want to dwell on her pain and would much rather participate in creative-outlet courses instead of pain management courses. She had tried both of these and had experienced that creative and artistic courses enhanced her quality of life to a much greater extent than did pain management courses. For several of the participants, the search for an answer continued. Even some of the participants who talked about accepting their health complaints kept the door open for other explanations. There were also participants who regarded the new filling materials with some skepticism.

And now I just heard that they have started talking about the new filling materials, the white ones, you know. Because there are people who react to those as well, you know.

I have almost nothing like that, because I mostly have, uhm, porcelain crowns, you know. That was a conscious choice I made at the time. However, I have no idea what they used to cement the crowns.

The not-knowing part of their health complaints seems to have made acceptance and management of the complaints difficult. The majority of the participants had other diagnoses, or went on to receive other diagnoses, explaining part of their health complaints. When describing the management of these complaints, including potentially life-threatening adverse reactions to prescribed medication, participants seemed less emotionally engaged than when describing suffering from the complaints they could neither explain nor knew how to treat.

## Discussion

The opening phrase in the interviews was formulated along the lines: “The main focus for this interview is possible changes in health complaints and quality of life after amalgam removal. However, we do know that things in life are connected, so we are interested in the big picture.” We thereby opened for a broad understanding of what was meant by “after amalgam removal” because “after” could be understood either as “in the period following” or as “caused by.” In their answers, participants seemed to alternate between these interpretations. When they became aware of this, they tried to sort out what was reasonable to connect with the dental amalgam and what might be related to other things. Most participants stressed how difficult these were to untangle and how it was impossible to make strong claims. Through the participants’ descriptions, a pattern emerged of “searching for an answer, trying out a solution, and evaluating the effect.” The majority of the participants described having been through similar circular procedures of searching for an answer, trying out a solution, and evaluating the effect before the amalgam removal, and some described having started on new searches after the removal.

When drawing conclusions, one is always at risk of accentuating some aspects of participants’ experiences over others. In our interview material, the energy and drive the participants put into their search for a diagnosis and a cure really stand out. It could be argued that this automatically follows from the experienced discomfort; however, the participants seemed to invest the same drive and energy in taking care of their families and their work obligations. The majority of the participants seemed to hold themselves to quite high standards and they expressed both sadness and frustration over not being able simply to “pull themselves together.” Through these descriptions, we were able to glimpse a sense of despair and chaos; however, this was often quickly brushed aside with a curt laugh, a joke, or a shift in focus.

According to Cassell (1982, p. 640), “suffering occurs when an impending destruction of the person is perceived; it continues until the threat of integration has passed or until the integrity of the person can be restored in some other manner.” In more general terms, Cassel defined suffering as “the state of severe distress associated with events that threaten the intactness of the person” (Cassell, 1982, p. 640). Consequently, it is not only the pain and the health complaints in and of themselves that are important, but also the perceived implications these have for the individual's everyday life, hopes for the future, and sense of self. In our interview material, the “threat to the intactness of the person” seems mostly to have been associated with participants’ being unable to fulfill their obligations as employees and family members.

Despite this complexity, we find that “pain” and “suffering” are often used interchangeably in everyday language. This is not a trivial distinction and to treat it as such can potentially lead to more suffering. According to Loeser (2000), it is the suffering, and not the pain, that motivates people to seek medical care. Nevertheless, it is usually the pain, or the health complaints, which are addressed by both the patient and the physician. If patients seek relief for their suffering, which they perhaps are not even able to distinguish from their pain, and doctors are trained to diagnose and treat pain and/or health complaints, it is hardly surprising that patients with unexplained health complaints often describe their encounters with the medical profession as far from satisfactory.

As argued by Kirmayer (1994, p. 183), suffering can be transformed by the meanings given to the experienced symptoms and distress. In continuation of this, he says that to be effective—that is, “to carry private conviction and rhetorical force” (p. 184)—the illness meaning must be perceived as having some sort of authority. Within a biomedical understanding of illness and disease, authority is generally granted through a diagnosis. As summarized by Jutel (2010, p. 229), a “medical diagnosis explains, legitimizes, and normalizes.” In the absence of a diagnosis, patients are denied an explanatory framework through which they can understand, and potentially give meaning to, their complaints. It should therefore not come as a surprise that many patients consider a diagnosis as a prerequisite for finding meaning and restoring “the integrity of the person” (Cassell, 1982, p. 640). For many patients, including our patient group, a single diagnosis by which all complaints can be explained cannot always be obtained. This leaves the patients with more unknowns than answers: Where are they supposed to direct their energy? Can they trust that their complaints will stay more or less stable, or do they have to anticipate getting worse? Should their efforts be focused on adapting and coping, or should they continue searching for an explanation and a cure? How can they integrate their sense of self with their (new) everyday life?

One thing that seemed to be of importance for all our participants, with all their similarities and differences, was the fact that they were all very happy to have had all their amalgam fillings removed. They were, however, unwilling to state unequivocally that they had become better because of the amalgam removal, and the majority seemed to lean toward the hypothesis that amalgam removal played a part along with all the other changes in their lives. Participants sometimes during the interviews referred to more simplistic convictions; these were, however, quickly contrasted with more complex and open-ended explanations. Different explanations seemed to be accompanied by different levels of emotions and rationales. Some of the most important aspects of the amalgam controversy are perhaps found in the difference between the rational understanding of multifactorial explanations of health and the emotional activation seen when a participant imagines having one amalgam filling left. This underscores how important it is that both researchers and health personnel learn more about how patients think, act, and feel regarding these questions.

Several of the participants in our sample seemed to construe the amalgam removal as a prerequisite enabling them to start the process of accepting their health complaints. Without it, they feared they would have continued to worry that their amalgam fillings stood between them and good health. Nevertheless, our participants were also quick to point out that for most of their health complaints, they could not be certain that these were causally linked to their amalgam fillings. It is reasonable to assume that the emotional side of the question “Are my amalgam fillings making me ill?” is often left out of the medical encounters, or perhaps it is only answered by referring to statistics and probabilities. Even though health personnel and researchers might find comfort in, and take guidance from the evidence indicating that dental amalgam is a safe treatment option at group level; the same evidence, with its corresponding statistical and clinical uncertainties, does not necessarily sound equally convincing to the patients who are trying to figure out whether it is true for their lives.

For some patients, it would perhaps be beneficial to be able to address these issues based not only on general probabilities but also on the direct consequences the complaints and the uncertainties linked to the dental amalgam have in their life. It is our strong belief that taking the time to address this would be an important step toward addressing not only the pain but also the suffering and fear related to the pain. For some patients, this could result in their being better able to live with their health complaints and the uncertainties related to the origin and prognosis of the complaints. For other patients, the worry deriving from their dental amalgam could potentially still have a too negative impact on their quality of life.

When considered in light of stories of successful recoveries in the media, patients’ continued wish to have their amalgam fillings removed does not appear unreasonable. Several studies have reported that patients experience improved health after amalgam removal (Lygre et al., 2005; Melchart et al., 2008; Nerdrum et al., 2004; Sjursen et al., 2011). This has also been described in the qualitative studies performed within this field (Jones, 2004; Stahlnacke & Soderfeldt, 2013). It has been difficult, however, to pinpoint the exact causes for the reported health improvements, and the patients’ health complaints have not been reduced to such an extent that they have reached the levels of health complaints found in the general population.

The fact that we do not fully understand the reason for the reported improvements is perhaps most disconcerting for the researchers and the health professionals. For many patients, a subjective perception of reduced health complaints will have its own value irrespective of the mechanisms involved. In continuation of this, it could be argued that it should be easier for patients to have all their amalgam fillings removed. However, removal of dental amalgam should never be considered a treatment if other possible causes for the complaints have not yet been ruled out (Norwegian Directorate of Health, 2008). In addition, there will always be risks associated with removing sound dental amalgam fillings. These risks must be appropriately described by the dentist before amalgam removal is initiated (Norwegian Directorate of Health, 2008).

### Reflexivity, scope, and limitations

The cross-disciplinary approach of this study enabled us to look at the patients’ experiences from different clinical angles; however, there is also a risk that our clinical stance could overshadow the perspectives of the patients. At the participants’ first examination at the specialty unit, no objective findings (i.e., contact allergic reactions) of adverse reactions to dental amalgam were found, and it was not recommended that the participants have their dental amalgam removed. This also meant that they could not have the cost of the amalgam removal covered by social security. In the interviews, the participants expressed a strong wish to have their fillings removed, but except for making sure that defective fillings were replaced with other materials than dental amalgam, no one had initiated a full amalgam removal on their own. This could be because they were relatively reassured by the examination and the advice from the specialty unit, or it could be because of lack of financial means. From the interviews, we get the impression that both explanations played a part. Therefore, we have to assume that our participants were not among the most strongly convinced anti-amalgam patients, and our findings have to be interpreted accordingly.

When interpreting our findings, it is also important to take into consideration that the participants had taken part in a treatment study for which the aim was to investigate the effects of amalgam removal, and that they were told in advance that changes in health complaints after amalgam removal would be the topic in the interviews. To reduce the impact of links to the clinical trial, interviews were carried out at a different location than the follow-ups. Moreover, the interviewer had not been part of the follow-ups. It soon became clear that the interviewer was nevertheless considered a member of the specialty unit.

The participants might also have reacted to subtle cues from the interviewer, perhaps unintentionally prompting multifactorial explanations at the expense of other explanations. The fifth author, who listened in on the interviews, had the impression that different explanations were met with equal interest. The participants, however, might have experienced this differently. It is reasonable to assume that the topic and context of the interviews might have accentuated our finding that patients seemed to be more worried about the health complaints that they could not explain and which could potentially have been caused by the dental amalgam, than by pain and health complaints caused by other diagnosed medical conditions.

Interviews were performed 5 years after removal of dental amalgam. The explanations and descriptions given in the interviews would have been different if the interviews had taken place before or shortly after the amalgam removal. However, the aim of the exploration presented in this article was to learn more about how participants experienced and gave meaning to changes in health complaints before, during, and after amalgam removal, and not to obtain an exact chronological description of every experience. The stories related by the participants are the stories they live with, the stories through which they remember and give meaning to their experiences.

## Conclusion

If patients’ experiences 5 years after amalgam removal can be summarized in a single sentence, the following might be appropriate: “The dental amalgam was certainly important to get rid of, but it is uncertain how important the removal was for the experienced changes in health complaints.” Patients were very happy to have had all their amalgam fillings removed, but they did not believe that they could credit all the positive changes to the amalgam removal. Nevertheless, several of the participants said that the amalgam removal had been very important because it meant that they could cross this particular worry off the list. For some participants, this also meant that they thought they might be moving toward a personal acceptance of their health complaints.

## Ethics and consent

The project was approved by Regional Committee for Medical and Health Research Ethics in Western Norway (REK III nos 24.01 and 2007/10173-ARS), and the Norwegian Social Science Data Services (NSD 19306).
